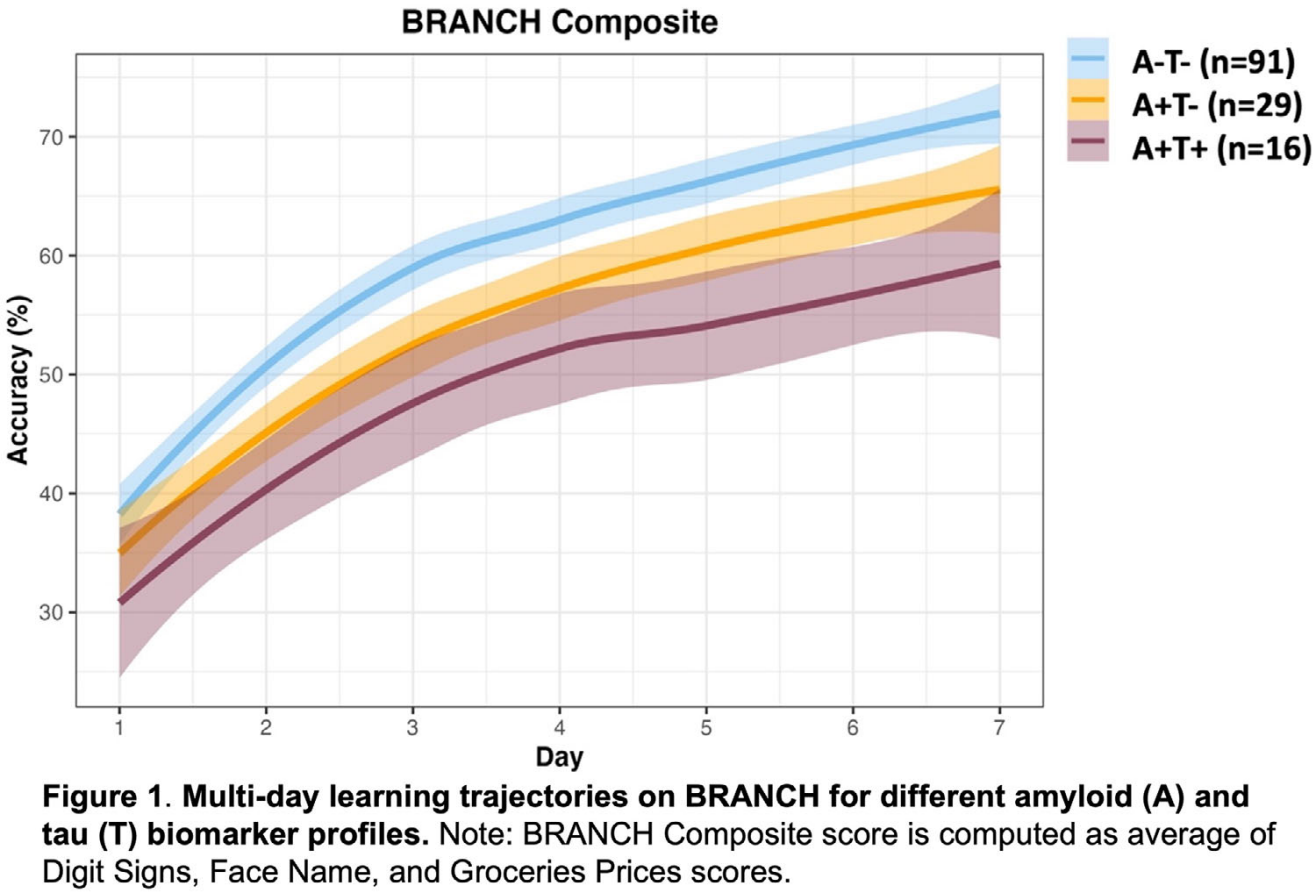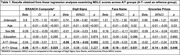# Leveraging a multi‐day learning paradigm to reveal early amyloid‐ and tau‐ related cognitive deficits in preclinical Alzheimer’s disease

**DOI:** 10.1002/alz.086313

**Published:** 2025-01-03

**Authors:** Roos J Jutten, Daniel Soberanes, Cassidy Molinare, Stephanie Hsieh, Michelle E. Farrell, Dorene M Rentz, Gad A Marshall, Keith A Johnson, Reisa A Sperling, Rebecca E Amariglio, Kathryn V Papp

**Affiliations:** ^1^ Massachusetts General Hospital, Harvard Medical School, Boston, MA USA; ^2^ Brigham and Women’s Hospital, Harvard Medical School, Boston, MA USA

## Abstract

**Background:**

Individuals with preclinical Alzheimer’s disease (AD) show reduced practice effects on annually repeated neuropsychological testing, suggesting a decreased ability to learn over repeated exposures. Remote, digital testing enables the assessment of learning over more frequent time intervals, thereby facilitating a more rapid detection of those early learning deficits. We previously showed that multi‐day learning on the Boston Remote Assessment for Neurocognitive Health (BRANCH) was indeed diminished in Αβ+ cognitively unimpaired (CU) older adults. Here, we further investigated the impact of tau pathology on BRANCH multi‐day learning curves (MDLCs).

**Method:**

N = 136 CU older adults (age = 73.4±7.6, 66% female, 16.6±2.4 years education) from three well‐characterized cohorts completed multi‐day BRANCH on their personal device. The assessment includes two associative memory tests (Face Name and Groceries Prices) and a processing speed test with an associative memory component (Digit Signs) with identical stimuli repeated for seven consecutive days. An MDLC score is computed using an area under the curve method allowing for the combination of day 1 performance with a non‐linear learning trajectory over the subsequent six days. All participants had [11C]Pittsburgh compound‐B and [18F]flortaucipir PET within 0.7±0.5 years of BRANCH and were classified as A ± (global amyloid burden, DVR cutoff 1.14) and T ± (inferior‐temporal tau SUVr, cutoff 1.30), resulting in n = 91 A‐T‐, n = 29 A+T‐ and n = 16 A+T+. Linear regression analyses adjusting for age, sex, and education were used to examine differences in BRANCH day 1 and MDLC scores across A/T groups.

**Result:**

No A/T group differences were detected using day 1 scores. However, MDLC scores increasingly diminished across groups, with the A+T‐ group performing marginally worse (ß = ‐0.04,95%CI[‐0.08–0.01], p = 0.11) and the A+T+ group significantly worse (ß = ‐0.06,95%CI[‐0.11–0.01], p = 0.03) than A‐/T‐ (Figure 1). A+ status regardless of T‐status was associated with diminished Digit Signs MDLCs, whereas being T+ drove worse performance on Face Name and Groceries Prices MDLCs (Table 1).

**Conclusion:**

Subtle differences in learning among CU older adults with different A/T biomarker profiles are observable using MDLCs. These findings further support the notion that a multi‐day learning paradigm can provide unique information about cognition that is not captured using a single time‐point assessment and is particularly relevant in preclinical AD.